# A Multidisciplinary Approach to Rhinoplasty: The Facial Plastic Surgeon and the Psychologist

**DOI:** 10.1055/a-2741-2458

**Published:** 2025-11-17

**Authors:** Deborah Auer, Sarup Saroha, Samit N. Unadkat, Hesham A. Saleh

**Affiliations:** 1 Charing Cross Hospital, London, United Kingdom; 2 University College London, London, United Kingdom; 3 Royal National ENT and Eastman Dental Hospitals, London, United Kingdom; 4 Imperial College London, London, United Kingdom

**Keywords:** rhinoplasty, plastic surgery, psychology, body dysmorphic disorders, multidisciplinary team

## Abstract

**Introduction:**

Rhinoplasty is one of the most frequently requested aesthetic procedures. However, a subset of patients presents with complex psychological profiles that can adversely impact surgical outcomes. Early psychological assessment is crucial to optimizing patient safety and satisfaction.

**Objectives and Hypotheses:**

This narrative review aims to explore the role of integrated psychological assessment within multidisciplinary facial plastic surgery services. It hypothesizes that early psychological input improves patient selection, manages expectations, and reduces revision rates.

**Study Design:**

Narrative review.

**Methods:**

We conducted a structured narrative review of PubMed/MEDLINE, PsycINFO, Embase, and Scopus (from inception to June 2025), supplemented by clinical experience from a high-volume United Kingdom rhinoplasty center. Search terms included rhinoplasty, psychology, BDD, screening, and multidisciplinary care. Eligible sources comprised peer-reviewed studies, reviews, and guidelines on psychological assessment or outcomes in aesthetic rhinoplasty.

**Results:**

Integrated psychological assessment can identify patients at risk of dissatisfaction, enhance patient selection, reduce revision surgeries, and improve overall outcomes.

**Conclusion:**

Multidisciplinary collaboration between surgeons and psychologists improves rhinoplasty outcomes, enhances patient safety, and supports the ethical principle of nonmaleficence.

## Introduction

Rhinoplasty is one of the most commonly requested elective facial plastic procedures worldwide, performed for both aesthetic and functional indications. 1 2 3 Its central role in reshaping facial harmony and improving nasal airflow makes it a cornerstone of modern facial plastic surgery.

While surgical technique remains critical, a growing body of literature and clinical experience highlights the psychological complexity of patients seeking rhinoplasty. Many individuals present with long-standing dissatisfaction with their nasal appearance, often originating in adolescence. This concern may be deeply intertwined with core aspects of their identity and self-image. Patients may then be deeply emotionally invested in the procedure, and if outcomes fall short of expectations, dissatisfaction may ensue despite objectively successful surgical results. 4

Historically, a subset of patients presenting for rhinoplasty has exhibited comorbid mental health conditions, prompting psychological referral to assess surgical suitability. The biopsychosocial model supports the integration of psychological, social, and biological factors in health, underscoring the value of involving psychologists in both preoperative assessment and postoperative care. 5 6 7 Such multidisciplinary collaboration improves patient outcomes and may reduce revision rates and long-term healthcare burdens.

This narrative review explores the integration of psychological care within a facial plastic surgery service, focusing on rhinoplasty. It highlights the value of psychological input throughout the patient journey, outlines indicators for referral, and provides practical guidance for incorporating preoperative assessment into routine care. Drawing on institutional experience, it advocates for a structured multidisciplinary model to improve patient selection, safety, and long-term satisfaction.

## Methods

This study presents a structured narrative review, combining published evidence with clinical insights from a high-volume United Kingdom facial plastic surgery center. The aim was to synthesize current knowledge on psychological assessment in rhinoplasty and provide practical guidance for multidisciplinary implementation.

A literature search was conducted across four databases: PubMed/MEDLINE, Embase, Scopus, and PsycINFO, covering records from database inception to June 30, 2025. Search terms included combinations of: “rhinoplasty,” “cosmetic surgery,” “psychological assessment,” “body dysmorphic disorder (BDD),” “screening,” “trauma,” “multidisciplinary,” “mental health,” and “patient selection.” Boolean operators and truncation were applied to adapt each query to database-specific syntax. Reference lists of key articles and relevant guidelines were also manually screened to identify additional sources.

Articles were included if they addressed psychological, psychiatric, or psychosocial aspects of aesthetic or revision rhinoplasty. Eligible sources encompassed original studies (quantitative or qualitative), systematic reviews, narrative reviews, practice guidelines, and professional consensus statements. We excluded non-English language articles, publications focused solely on surgical technique, opinion pieces lacking substantive content, and studies limited to nonaesthetic nasal surgery unless psychological variables were a central focus.

One reviewer screened all titles and abstracts for relevance. Full texts of potentially eligible articles were retrieved and assessed against the inclusion criteria. Key data were extracted on study design, population, psychological constructs, screening tools, and clinical recommendations. A thematic narrative synthesis was used to group findings conceptually, rather than through quantitative meta-analysis, due to the heterogeneity of included sources.

In addition to the published literature, this review draws on the clinical practices of a high-volume United Kingdom rhinoplasty service where routine psychological assessment is embedded in multidisciplinary care. No individual patient data were used, and no ethical approval was required.

While this review aimed for breadth and clinical relevance, the narrative design does not include a formal risk-of-bias assessment or quantitative synthesis. The findings should be interpreted in the context of methodological heterogeneity across included sources and the descriptive nature of the synthesis.

The thematic analysis drew upon a diverse range of sources, including original research studies, systematic and narrative reviews, clinical guidelines, psychological literature, and diagnostic manuals. Most references were published between 2000 and 2025, ensuring contemporary relevance while incorporating foundational psychological and ethical frameworks appropriate to the multidisciplinary context of aesthetic rhinoplasty.

## Why Psychology Matters in Rhinoplasty

As the most central and visually prominent facial feature, the nose plays a disproportionate role in perceived attractiveness, identity, and social confidence. 8 Thus, rhinoplasty is more than a technical procedure; it is intrinsically linked to a patient's self-perception, body image, and emotional well-being. Patients often seek surgery believing it will enhance not only their appearance but also their personality traits and even perceived age. 9 10 11 This places rhinoplasty within the category of body image surgery, where emotional investment and personal meaning may far exceed the physical correction proposed. Importantly, for some patients, psychological therapy may be more effective in helping them meet these internal goals than surgery itself. 12 13

Facial plastic surgeons often face the challenge of assessing not only physical suitability in the context of psychological readiness for surgery. When a patient presents with unrealistic expectations or difficulty processing potential risks and imperfections of rhinoplasty, referral to psychology becomes a key safeguard. Some surgeons describe a “gut feeling” against proceeding, an instinct that warrants attention, particularly given that revision rates in rhinoplasty may be as high as 16%. 14 This risk increases in patients who have already undergone procedures with multiple surgeons, 15 as each prior surgery may reflect a pattern of dissatisfaction or unaddressed psychological distress. Exploring a patient's prior surgical journey and how they engaged with other clinicians can offer critical insight into how they might respond to further procedures.

According to Beauchamp and Childress' principles of biomedical ethics, the need for nonmaleficence, to do no harm, applies physically and also psychologically. 16 If there is concern that surgery could harm a patient's mental health or exacerbate underlying conditions such as body dysmorphia or trauma, the procedure should be deferred pending a psychological assessment. Historically, medical practice has drawn a false divide between body and mind, but modern multidisciplinary care increasingly identifies the need to treat both in tandem. 12 Collaboration with psychologists ensures that surgeons can select their patients appropriately, to provide holistic care that reduces the risk of harm and enhances long-term satisfaction. 8

## Key Psychological Indicators in Aesthetic Patients

Surgeons performing rhinoplasty should understand the psychological factors that influence patient selection and postoperative outcomes. In many cases, patients stand to benefit from psychological input, whether to support decision-making, manage expectations, or safeguard mental well-being. Communication between patient and surgeon is pivotal in determining postoperative satisfaction, 17 with much of the outcome shaped by the rapport established during consultations. 18 Recognizing psychological red flags early in the clinical encounter can help guide appropriate referrals and reduce the risk of poor outcomes.

### Depression

Depression is a common psychological feature among patients seeking aesthetic rhinoplasty. Studies have shown that these individuals often report higher levels of preoperative psychological distress compared with those undergoing rhinoplasty for functional reasons. 4 19 20 Depression may affect patient expectations, pain perception, and postoperative recovery, contributing to dissatisfaction even after technically successful outcomes. When depression is moderate to severe, it may represent a contraindication to surgery unless treated effectively. Identifying at-risk patients through careful clinical interviews and psychometric tools is crucial for ensuring safe patient selection.

### Appearance-Related Anxiety

Anxiety, particularly centered around body image, frequently coexists with depression. A psychologist's role is to determine whether the level of concern is proportionate or excessive, causing functional impairment. Appearance-related anxiety may present with safety behaviors such as heavy makeup use, frequent mirror checking, or avoidance of social situations. Red flags include patients who catastrophize minute discrepancies with millimeter precision, or who demonstrate difficulty engaging meaningfully with the informed consent process, such as struggling to retain information, asking repetitive reassurance-seeking questions, or appearing unable to appreciate the limitations and risks of surgery. These behaviors may signal deeper psychological distress or underlying psychopathology that could be exacerbated by surgery.

### Emotional Lability and Vulnerability in the Perioperative Period

Patients with emotional instability or high psychological reactivity may struggle to manage the uncertainties inherent in surgery. The perioperative period, including anesthesia, postoperative swelling, and uncertainty about final results, can intensify emotional responses. Those with a history of trauma may experience heightened anxiety or even re-traumatisation. 21 22 Psychologists can help assess a patient's resilience and readiness, identifying whether additional support is required before proceeding to surgery.

BDD features an excessive preoccupation with perceived flaws in appearance that are either minor or not observable to others, which causes significant shame and interference in the person's life. 23 It is the most commonly documented psychiatric condition among individuals seeking cosmetic surgery, affecting between 4 and 57% of cosmetic surgery patients, 24 compared with 1% in the general population. 25 26 For these patients, nasal appearance is the most common focus. 27 28 Despite this, BDD may go unrecognized as patients can present with articulate and seemingly rational concerns. Surgeons unfamiliar with the nuances of BDD may inadvertently select these patients, only to discover that surgery worsens psychological symptoms. Warning signs include shame-based narratives, relentless requests for revision surgery, and the inability to accept surgical risk or imperfection.

In some cases, body image concerns may originate from another individual, typically a parent, projecting their own insecurities onto the patient. This phenomenon, known as BDD by proxy, 29 can drive individuals to seek surgery they might not otherwise desire. If a parent or family member insists on surgery for a young adult or adolescent, clinicians should be alert to the risk of such pressure and explore family dynamics further.

Surgery rarely resolves the core cognitive distortions in BDD. In some cases, it exacerbates previously managed symptoms and leads to worsening anxiety, obsessive behaviors, or a shift in focus to other perceived flaws. 30 An early psychological referral is essential. Even if surgery proceeds later, the aim is to align patient expectations with realistic outcomes and reduce the emotional burden associated with surgical intervention.

Low self-esteem is common in patients seeking aesthetic rhinoplasty. Naraghi et al found that aesthetic patients scored lower on the Rosenberg Self-Esteem Scale than those undergoing surgery for functional reasons. 8 Individuals with low self-esteem may place unrealistic emotional weight on surgical outcomes, believing that aesthetic improvement will fundamentally alter their self-worth or social experience. Unsurprisingly, these patients are more prone to dissatisfaction, even if the surgical result is objectively favorable.

Conversely, other studies have indicated that women with higher self-esteem feel less interest in cosmetic surgery. 18 31

It is essential to distinguish between body dissatisfaction and body shame. While dissatisfaction refers to disliking a specific feature, shame encompasses feelings of inadequacy, vulnerability, and inferiority. Body shame is often rooted in early relational trauma or experiences that diminish self-worth, such as bullying or abuse. These emotional wounds can shape distorted internal narratives that surgery cannot resolve. For this reason, incorporating psychological assessment tools, such as self-esteem questionnaires, can help identify candidates who may require preoperative support or psychological therapy before considering surgery.

Early psychological trauma, particularly from adverse childhood experiences (ACEs), can significantly shape a patient's body image, self-worth, and motivations for seeking aesthetic surgery. This includes developmental or relational trauma, which arises from chronic emotional harm within early attachment relationships, such as neglect, misattunement, or abuse by caregivers. This form of trauma is deeply embedded in both mind and body, disrupting emotional regulation, body awareness, and one's internal sense of identity, even without a single identifiable traumatic event. 21 Operating on patients with unresolved relational trauma can lead to postoperative dissatisfaction, frequently driven by shame-based internal processes that surgery alone cannot resolve. These patients may unconsciously project unmet emotional needs onto the surgical journey, viewing the surgeon as a caregiver or rescuer figure. If the procedure fails to meet their expectations, the emotional fallout may be disproportionate to the objective outcome, leading to distress, mistrust, or repeated surgical requests. 18

Trauma also affects psychological resilience. Patients with a history of early relational trauma may have difficulty coping with uncertainty, complications, or imperfections in surgical results. 18 22 Assessing the impact of trauma requires specific training and should be approached with care. Clinicians must ask about past experiences in a nonthreatening, empathic manner, as poorly handled discussions risk re-traumatisation. 21 22 For this reason, psychological exploration of trauma should ideally be performed by a trained psychologist.

Surgeons should be cautious in proceeding with rhinoplasty in patients with known or suspected ACEs without a thorough psychological assessment. Identifying trauma-informed indicators before surgery allows for safer decision-making and may prevent adverse postoperative outcomes.

## Preoperative Psychological Assessment

Although psychological screening is increasingly recognized as best practice in aesthetic surgery, implementation remains inconsistent and may be left to the discretion of individual clinicians or institutions. 32 33 34 Australia stands out as an exception, introducing national guidelines in July 2023 that mandate psychological assessment for all patients undergoing cosmetic procedures, including rhinoplasty. 35

The primary goals of preoperative psychological assessment are to identify patients who may be at increased risk of postoperative dissatisfaction or harm due to unrealistic expectations, psychiatric comorbidities, or unresolved trauma. Psychological screening also plays a critical role in:

- Supporting informed consent by ensuring the patient can understand and retain information about potential outcomes and risks.
- Evaluating emotional readiness for surgery, including being able to tolerate ambiguity, imperfection, and delayed gratification.
- Facilitating timely referral to mental health professionals when psychological input is needed before proceeding.

Ultimately, these goals align with the principle of nonmaleficence, ensuring that surgery is not only technically appropriate but also emotionally and psychologically safe for the patient.

Psychological assessment explores three key domains:

- Motivation: whether the patient's desire for rhinoplasty is driven internally (e.g., improved confidence) versus externally imposed (e.g., coercion or unrealistic social ideals).
- Readiness: the patient's ability to manage the perioperative process, including recovery, transient swelling, and uncertainty about final results.
- Risk: screening for psychiatric diagnoses such as BDD, depression, anxiety, or trauma, all of which may impair decision-making or surgical satisfaction.

These dimensions provide a framework for distinguishing between patients likely to benefit from surgery and those who may require further psychological preparation.

Timing is critical. Assessments should ideally occur early in the preoperative journey, allowing sufficient time for further psychological input if necessary. Particular caution is needed in patients undergoing recent life instability, such as bereavement, relationship breakdown, or job loss, as these experiences may impair judgement or distort the underlying motivation for surgery. Similarly, patients with chronic unresolved issues, such as past trauma, may view surgery as a means of emotional repair rather than addressing the core psychological distress. When assessments are left too close to the surgical date, these vulnerabilities may go unrecognized or leave insufficient time for therapeutic intervention.

A combination of structured psychometric tools and clinical interviewing provides the most reliable approach. While validated questionnaires (see “Preoperative Psychological Assessment” section) offer objective metrics, they should complement, not replace, the nuanced insights gained through conversation and behavioral observation. A clinician's ability to detect discrepancies between what is said and how it is communicated (e.g., tone, body language) remains invaluable.

This preparatory phase sets the foundation for the practical pathways described in “Psychological Screening and Referral in Practice” section.

## Psychological Screening and Referral in Practice

**Fig. 1:**
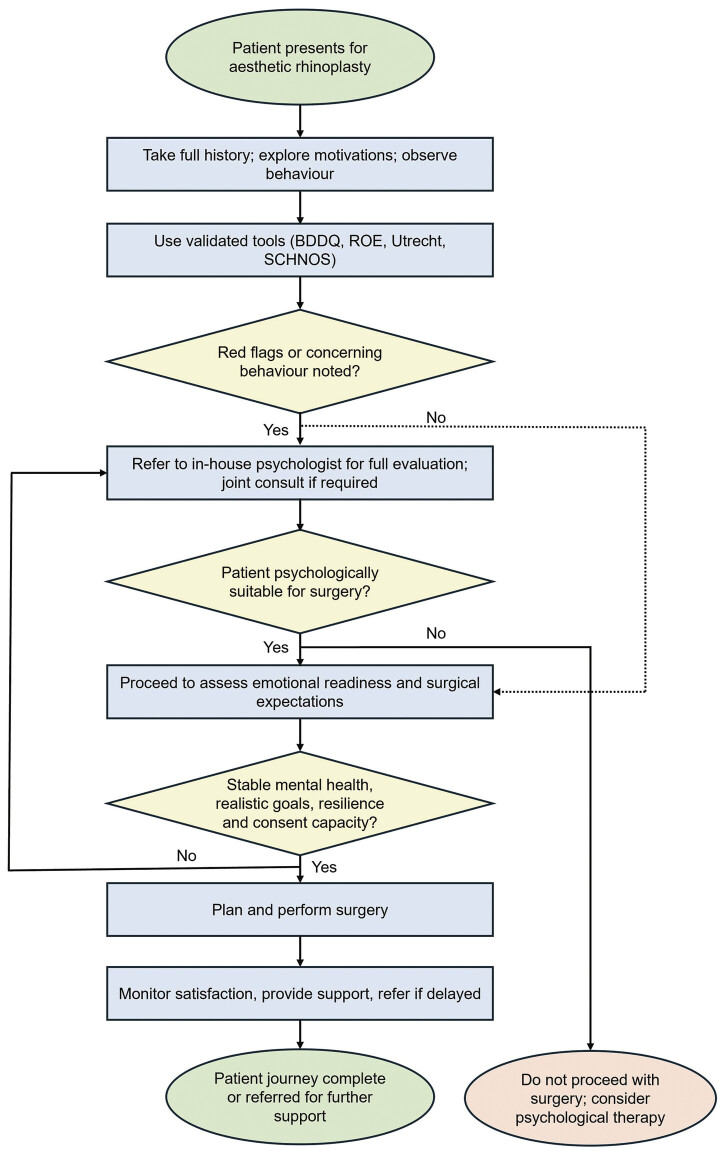
The flowchart outlines the stepwise process from initial consultation through validated screening, psychological referral if indicated, assessment of emotional readiness and surgical expectations, and final surgical decision-making, aiming to optimize patient safety, satisfaction, and outcomes.

At the authors' institution, psychological care is embedded within a formalized, multidisciplinary model of rhinoplasty delivery. A dedicated in-house psychologist is integrated into the facial plastic surgery team, ensuring that psychological evaluation is a standard component of the care pathway rather than an optional adjunct. This structure supports the early identification of patients at risk of poor outcomes, facilitates collaborative decision-making, and upholds ethical standards in aesthetic practice ( Fig. 1 ).

All revision and secondary rhinoplasty cases are automatically referred for psychological assessment, given the higher likelihood of prior dissatisfaction and underlying emotional complexity. For primary rhinoplasty patients, referral is more selective and based on clinical judgement or concerns identified during initial consultations. Red flags may include emotional instability, vague or shifting goals, appearance-related anxiety, or behaviors suggestive of trauma or BDD. In complex cases, joint consultations involving both the surgeon and psychologist are arranged to assess expectations, insight, and psychological readiness for surgery. These sessions often inform final decision-making and may require multiple appointments to ensure patient suitability.

To support this pathway, the service employs a range of validated screening tools before consultation. These include the BDD Questionnaire, 36 a brief but sensitive tool for identifying BDD, and the Rhinoplasty Outcome Evaluation, 37 which assesses satisfaction with nasal appearance and function. The Utrecht Questionnaire 38 is used to explore patient motivation and expectations, while the Standardized Cosmesis and Health Nasal Outcomes Survey 39 integrates both aesthetic and functional considerations. Together, these tools offer structured insight into the patient's psychological profile and help stratify the need for further assessment.

This model of integrated psychological screening and multidisciplinary collaboration has been instrumental in improving patient selection, minimizing risk, and enhancing both patient satisfaction and long-term outcomes.

In addition to structured screening tools, behavioral cues during consultation provide valuable insight into psychological readiness. Subtle interpersonal cues often reveal underlying psychological vulnerability. For example, eye contact can be highly informative: some patients may avoid sustained gaze, while others may exhibit what has been described as the “thousand-yard stare,” a distant, disengaged expression commonly associated with trauma-related dissociation. Hypervigilance is another key indicator; overly anxious or alert patients may struggle to process important preoperative information. In such cases, even a clear and empathetic explanation of risks may not be fully registered or retained.

Boundary testing may also manifest during the examination. Patients who recoil when touched, appear visibly distressed when their photographs are taken, or show signs of discomfort with routine clinical interactions may have unresolved trauma or attachment-related difficulties. Additionally, excessive preoccupation with communicating through medical secretaries or frequent, anxious outreach to staff can indicate a lack of containment and emotional regulation. Attention to grooming, punctuality, and the patient's ability to articulate their goals for surgery also contributes to the broader psychological picture. These behavioral observations, while subtle, should prompt further questioning and, where appropriate, psychological referral.

In aesthetic surgery, surgeons often describe an intuitive sense of discomfort when assessing certain patients, a “gut feeling” that something is not quite right, even without overt red flags. While difficult to quantify, this instinct frequently stems from inconsistencies in patient communication, vague surgical goals, or disproportionate emotional investment in the anticipated outcome. Surgeons may notice a mismatch between the patient's verbal enthusiasm and nonverbal cues, such as ambivalence, agitation, or excessive dependence.

Equally concerning are patients who present with a history of multiple prior cosmetic procedures, particularly when performed by different surgeons. In such cases, repetitive surgery may serve as a form of maladaptive coping or self-injury, a dynamic which places the current surgeon in the precarious position of becoming the latest “agent” in a psychologically driven cycle. If the surgeon experiences unease or hesitation despite the technical feasibility of the case, it is often safer to pause and initiate a psychological review before proceeding.

The behavioral and intuitive indicators outlined above may be easily overlooked in routine clinical settings. However, when systematically assessed, they provide powerful insights into a patient's psychological readiness for surgery. Table 1 summarizes key consultation-based cues that may warrant psychological input, allowing facial plastic surgeons to take a more holistic and preventative approach to patient care.

**Table 1:** Consultation-based behavioral cues and intuitive warning signs indicating psychological vulnerability in aesthetic rhinoplasty patients

Domain	Observation	Interpretation/clinical concern
Eye contact and facial engagement	Avoidant gaze, blank or “thousand-yard stare”	May reflect emotional dissociation or unresolved trauma
Hypervigilance	Overly anxious, highly alert, unable to focus or retain information	Risk of impaired consent and emotional dysregulation
Reactivity to examination	Recoiling from touch, discomfort with photography	Boundary issues, trauma history, or heightened shame sensitivity
Interpersonal boundaries	Excessive or inappropriate contact with staff (e.g., repeated calls to nurses or secretaries)	Poor containment, emotional dependency, or attachment difficulties
Self-care and presentation	Excessive grooming, heavy makeup, or attempts to conceal specific features	This may indicate body image preoccupation, appearance-related anxiety, or Body Dysmorphic Disorder
Clarity of surgical goals	Vague, shifting, or unrealistic expectations of outcome	Reflects difficulty with self-concept, poor insight, or magical thinking
Resilience and self-esteem	Grandiose or self-shaming language; little tolerance for imperfection	Suggests unstable self-image and low emotional resilience
Surgical history	Multiple prior cosmetic procedures, often with different surgeons	Possible compulsive behavior or underlying Body Dysmorphic Disorder
Surgeon's intuition	Persistent unease despite apparent suitability	May reflect nonverbal or relational cues signaling deeper psychological issues

## Conclusion

Rhinoplasty remains one of the most psychologically complex procedures within facial plastic surgery. While most patients pursue surgery with appropriate expectations and motivations, a significant subset presents with underlying vulnerabilities that can compromise outcomes. This review synthesized contemporary literature and institutional clinical experience to highlight key psychological indicators, such as mood disorders, BDD, low self-esteem, and ACEs, that warrant early identification and multidisciplinary management.

Drawing from a structured narrative review of published evidence, this study underscores the role of psychological factors in influencing surgical outcomes. These findings support a compelling case for formalizing multidisciplinary collaboration within rhinoplasty services. Embedding psychological expertise into the care pathway allows for earlier intervention, more nuanced assessment, and more ethical decision-making. While such collaboration is already implemented in select high-volume centers, widespread adoption remains limited. Establishing standardized referral pathways, employing validated assessment tools, and fostering routine collaboration between surgeons and psychologists will help ensure safer, more patient-centered care, particularly in revision or psychologically complex cases. A formalized multidisciplinary model aligns with principles of holistic care and may ultimately improve patient satisfaction, reduce revision rates, and protect both patients and practitioners from the unintended consequences of misaligned expectations.
